
JOURNAL INFORMATION
==============================
NLM Title Abbreviation: Curr Med Sci
Journal ID: Curr Med Sci
NLM Journal ID: 101729993

Current Medical Science

ISSN: 2096-5230
EISSN: 2523-899X

ARTICLE INFORMATION
==============================
PMCID: PMC7801315
PMID: 33351164
DOI: 10.1007/s11596-020-2279-9
Article ID: 2279
Article version: 1
Subjects: Erratum

Erratum to: Voltage-gated Sodium Channels and Blockers: An Overview and Where Will They Go?

Li Zhi-mei 1
Chen Li-xia 2
Li Hua 1
1 grid.33199.31 0000 0004 0368 7223 Hubei Key Laboratory of Natural Medicinal Chemistry and Resource Evaluation, School of Pharmacy, Tongji Medical College, Huazhong University of Science and Technology, Wuhan, 430030 China
2 grid.412561.5 0000 0000 8645 4345 Wuya College of Innovation, Key Laboratory of Structure-Based Drug Design & Discovery, Ministry of Education, Shenyang Pharmaceutical University, Shenyang, 110016 China
Electronic publication date: 2021 Jan 11
Print publication date: 2020
Volume: 40
Issue: 6
First page: 1206
Last page: 1206
Copyright: © The Author(s) 2020
License: This article is licensed under a Creative Commons Attribution 4.0 International License https://creativecommons.org/licenses/by/4.0/), which permits use, sharing, adaptation, distribution and reproduction in any medium or format, as long as you give appropriate credit to the original author(s) and the source, provide a link to the Creative Commons licence, and indicate if changes were made. The images or other third party material in this article are included in the article’s Creative Commons licence, unless indicated otherwise in a credit line to the material. If material is not included in the article’s Creative Commons licence and your intended use is not permitted by statutory regulation or exceeds the permitted use, you will need to obtain permission directly from the copyright holder. To view a copy of this licence, visit http://creativecommons.org/licenses/by/4.0/.
License URL: https://creativecommons.org/licenses/by/4.0/

Erratum for: Voltage-gated Sodium Channels and Blockers: An Overview and Where Will They Go? 39 6 16 12 2019 863 873 Curr Med Sci 10.1007/s11596-019-2117-0 31845216
issue-copyright-statement: © Huazhong University of Science and Technology 2020

==============================
The original article can be found online at 10.1007/s11596-019-2117-0

